# Comprehensive antigen profiling predicts post-surgical neuropathic pain in women treated for breast cancer

**DOI:** 10.1038/s41598-026-41637-6

**Published:** 2026-03-03

**Authors:** Helle Sadam, Laura Mustonen, Annika Rähni, Janne K. Nieminen, Maarja Toots, Mariliis Uusväli, Arno Pihlak, Pentti J. Tienari, Hanna Harno, Kaia Palm, Eija Kalso

**Affiliations:** 1 Protobios Llc, Tallinn, Estonia; 2 Department of Anaesthesiology, Intensive Care and Pain Medicine, University of Helsinki and Helsinki University Hospital, Helsinki, Finland; 3 Neurocenter, Neurology, University of Helsinki and Helsinki University Hospital, Helsinki, Finland; 4 Department of Chemistry and Biotechnology, Tallinn University of Technology, Tallinn, Estonia; 5 Translational Immunology Research Program, University of Helsinki, Helsinki, Finland

**Keywords:** Neuropathic pain, Post-surgical pain, Antibody response, Herpesvirus, Cancer, Diseases, Immunology, Microbiology

## Abstract

The importance of neuroimmune interactions in neuropathic pain (NP) has been established, but antibody-mediated mechanisms remain underexplored. In this explorative case-control study, we analyzed antibody profiles in patients with intercostobrachial nerve injury during breast cancer (BC) surgery. We compared 27 patients who developed chronic NP with 30 who remained NP-free, despite similar nerve injury. Plasma samples were collected before surgery and 4–9 years later. Mimotope variation analysis (MVA), a next generation random peptide phage display method revealed highly individual yet shared antigen profiles. We identified 1882 antibody epitopes differing between the study groups and that were associated with 79 common human pathogens. NP patients showed elevated pre-surgical antibody responses to viral epitopes of CMV (cytomegalovirus), EBV (Epstein-Barr virus), human papilloma virus-16 (HPV-16), human rhinovirus C3 (HRV C3), Herpes Simplex-1 (HSV-1), Herpes Simplex-2 (HSV-2), while antibody levels against Coxsackievirus B3 (CVB3) were lower. These findings persisted over time. The combination of responses to five viral epitopes (CVB3, EBV, CMV, HPV-16, HSV-2) predicted persistent NP (AUC 0.9, 95% CI 0.794–0.963). These findings implicate elevated antiviral immune responses in NP pathogenesis and encourage further clinical and basic research on the molecular mechanisms and novel treatment strategies for managing NP.

**Supplementary Information:**

The online version contains supplementary material available at 10.1038/s41598-026-41637-6.

## Introduction

Neuropathic pain (NP) is defined as pain caused by a lesion or disease of the somatosensory nervous system^1^. Surgical nerve injuries can lead to persistent peripheral NP (chronic post-surgical NP, CPSNP). CPSNP is especially prevalent (14–31%) in patients operated on for breast cancer (BC)^2,3^, injury to the intercostobrachial nerve (ICBN) during axillary surgery being an important cause. However, not all ICBN injuries lead to CPSNP. The biological mechanisms explaining why similar nerve injuries become painful in some, but not in all, still need to be elucidated.

Development and maintenance of pain following the onset of peripheral nerve lesion is a complex process involving multiple peripheral and central mechanisms^4,5^. Extensive preclinical and growing clinical evidence shows that neuroimmune interactions play a key role in this process and involve both innate and adaptive immune systems^6–8^. However, antibodies are less studied in the context of NP, although it has been shown that antibodies and their complexes have the potential to induce nociceptor hyperexcitability through multiple mechanisms^9,10^. In the context of nerve injury, pre-existing antibodies derived from previous exposures to environmental pathogens^11^, and autoantibodies, could enhance nociception due to trauma-related events^9,10^. Also, tissue injury could lead to exposure of hidden antigens and the generation of autoantibodies with pro-nociceptive properties^12,13^. However, the role of humoral immune responses in painful peripheral nerve injury has largely remained unexplored and clinical studies are scarce.

In this explorative study, we used mimotope variation analysis (MVA)^14^, a next generation random 12-mer peptide phage display method, for large-scale assessment of the antibody epitope repertoire in CPSNP after BC surgery, which had been complicated by nerve injury. The data generated by MVA immunoprofiling have successfully been used to identify novel antibody epitope biomarkers for vaccination-induced narcolepsy, multiple sclerosis, and COVID-19^14–17^. We compared the targeted peptide epitope profiles (immunoprofiles) in the clinical samples of patients with persistent painful versus painless surgical injuries to ICBN. In this, our aim was to assess whether specific immunodominant antibody epitopes associate with CPSNP after BC treatments. We analyzed samples from two timepoints: before surgery and at follow-up (4–9 years from surgery). In these settings, we addressed two questions: (1) Do patients with painful and painless surgical nerve injury present with a pre-existing distinct antibody epitope spectrum before nerve injury? (2) Are there CPSNP-associated changes in the immunodominant antibody response that emerges after surgical nerve injury and BC treatment? With this approach, our aim was to increase understanding of the immunological features in the pathophysiology of CPSNP and identify novel biomarkers for CPSNP.

## Results

### Patient characteristics

**Fig. 1:**
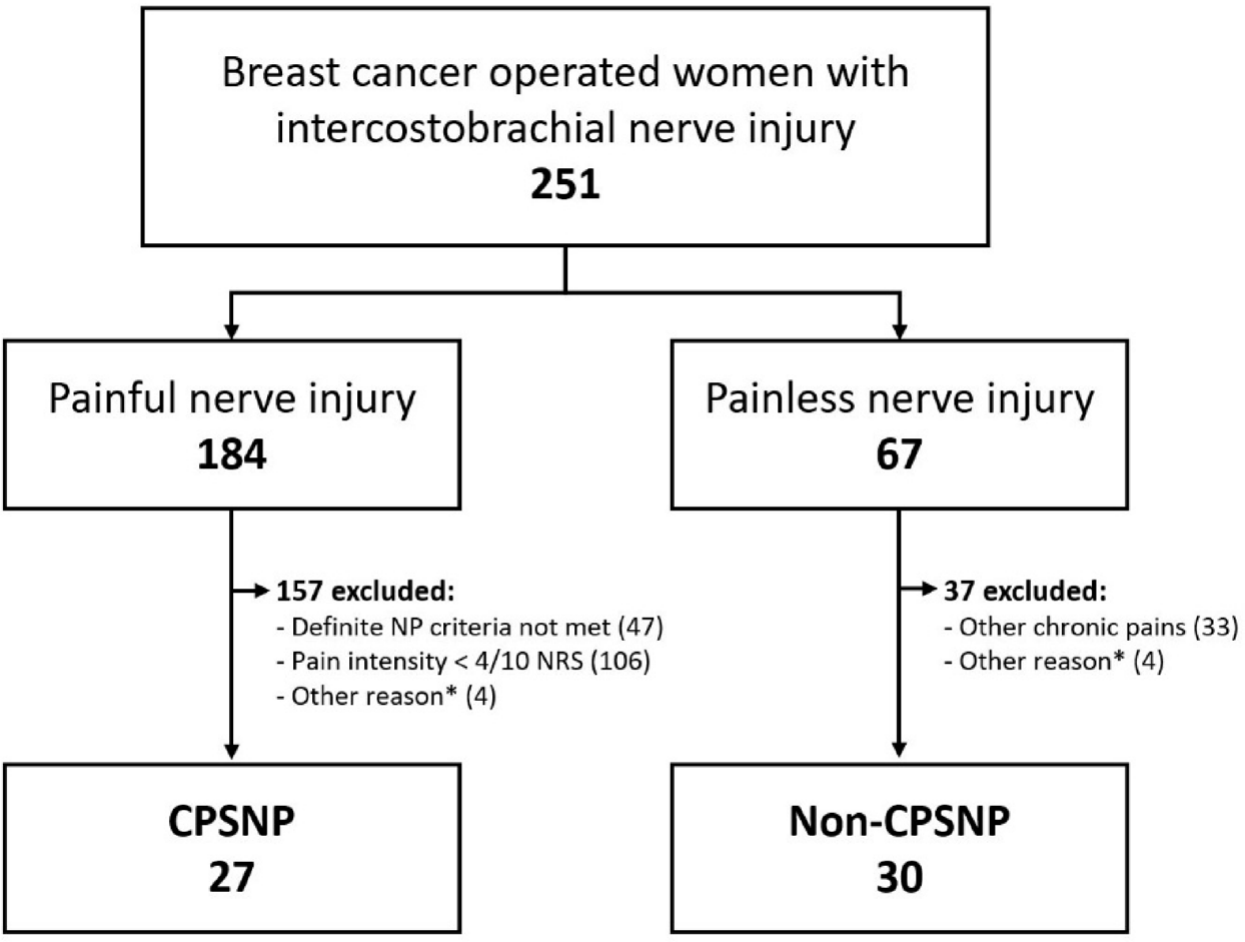
Patient selection. *Other reasons include metastasized cancer and diagnosis of inflammatory or neurological disease. CPSNP: persistent post-surgical neuropathic pain; NP: neuropathic pain; NRS: numerical rating scale.

**Fig. 2:**
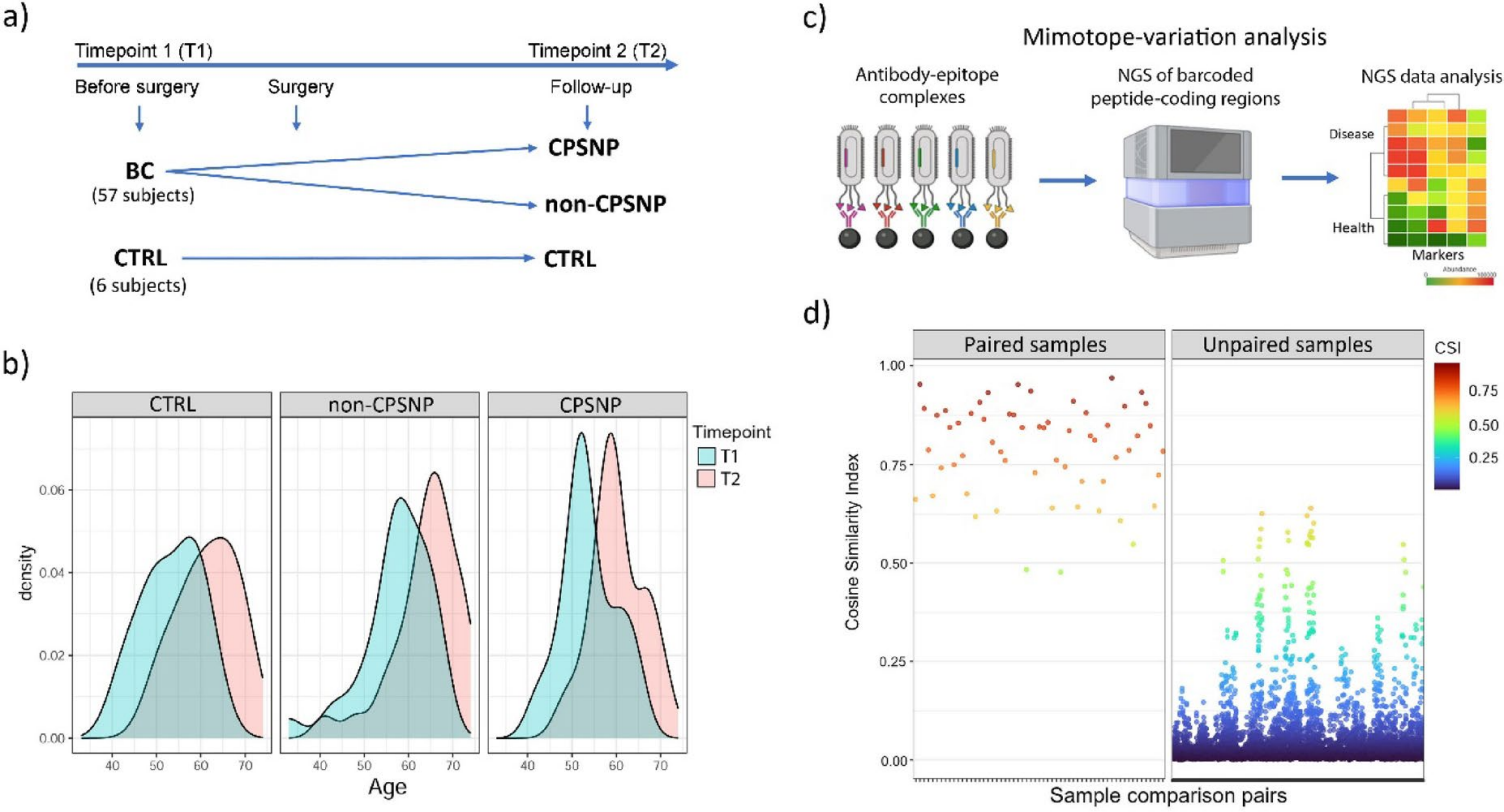
Characterization of antibody epitope profiles from the serum of patients with CPSNP (*n* = 27) and non-CPSNP (*n* = 30) by MVA. (**a**) Clinical study design. Samples from 57 patients with breast cancer (BC) diagnosis were analyzed. Each patient had given two samples: one before (timepoint 1, T1) and another after (timepoint 2, T2) surgery. The period between T1 and T2 varied between 4 and 9 years. The control group (CTRL) included serum samples derived from T1 and T2 timepoints from six patients from the same cohort without ICBN injury and without pain. (**b**) Age distribution of the 63 patients at T1 and T2. (**c**) Antibody epitope profiling with MVA. MVA workflow comprised different sequential steps: (i) immunocapture of IgG-phage complexes from a sample using the phage library (random 12-mer peptide M13 phage library); (ii) high-throughput Illumina HiSeq2500 short DNA sequencing of bar-coded phage DNA; (iii) bioinformatical data analysis resulting in antibody epitope identification. The sequenced DNA reads obtained were quality-checked, translated to peptide sequences, and sample- and cohort-specific epitopes from peptide sets were developed by the SPEXS2 pattern search algorithm. Schematic presentation was created with biorender.com (**d**) Highly individual immunoreactive epitope profiles shared similar features in paired sera (T1 and T2) samples, as observed from MVA. The 5000 most IgG-bound (abundant) peptide values (read counts) from each sample were taken into immunoprofile similarity analysis. For all sample pairs, the normalized scalar products of peptide count vectors were calculated for the cosine similarity index (CSI, R package lsa). The CSI values depicted are 0.70 and above (color-coded scale on the right), showing highly correlated immune profiles. Based on CSI analysis, the data of one CPSNP, one non-CPSNP and two CTRL samples were removed from quantitative analyses (Table S1). Group sizes: Paired samples *n* = 59, Unpaired sample pairs = 6844. Sample pairs were combined from timepoint samples of cohort patients: CPSNP *n* = 26, non-CPSNP *n* = 29 and CTRL *n* = 4 (Table S1). BC: breast cancer; CPSNP: chronic post-surgical neuropathic pain; CSI: cosine similarity index; CTRL: control; MVA: mimotope variance analysis; NGS: next generation sequencing; NP: neuropathic pain; T1: timepoint 1; T2: timepoint 2.

We used the same study group that was previously described by Lötsch et al.^18^ and Mustonen et al.^19^. We included 57 patients, of whom 27 had CPSNP, with a matched control group of 30 non-CPSNP patients (Fig. 1 and Table S1). The patient characteristics, including the type of cancer and cancer treatments, have been previously reported elsewhere^19^. At the follow-up visit, patients in the CPSNP group were on average approximately four years younger (mean age 60,3 vs. 64.0 years) than patients in the non-CPSNP group (Fig. 2b).

The groups had no significant differences in surgical and cancer treatments or in cancer type. Mastectomy was performed on 16 (59%) patients in the CPSNP group and 20 (67%) patients in the non-CPSNP group. Most patients (96% in CPSNP group and 83% in non-CPSNP group) had undergone axillary lymph node clearance. Chemotherapy was administered to 23 (85%) patients in the CPSNP group and 25 (83%) patients in non-CPSNP group. Docetaxel was included in the chemotherapy regimen in all but one patient in CPSPN and two patients in non-CPSNP group. Presurgical samples were taken from both groups (Timepoint 1 [T1]); the mean follow-up time was 6.5 years, ranging from 4 to 9 years (Timepoint 2 [T2], Fig. 2a). In the CPSNP group, the median worst pain past week score was 5/10 NRS, with overall pain intensity ranging from 4 to 8/10 NRS. For CPSNP patients, the median score in the Douleur Neuropathique 4 (DN4) screening questionnaire was 6/10 ranging from 3 to 8/10. At the follow-up visit, four patients in the CPSNP group reported on-demand use of paracetamol or NSAID, one patient reported on-demand use of paracetamol-codeine combination drug, and one patient used regular neuropathic pain medication (gabapentinoid and amitriptyline). None of the patients in the non-CPSNP group reported any use of pain medications. None of the patients had received any neuromodulatory pain treatments.

### The most abundant antibody epitopes are uniquely individual and consistently stable

To identify the antibody epitope response repertoire, we performed MVA immunoprofiling of serum samples from CPSNP and non-CPSNP groups collected at two timepoints (*n* = 126; Fig. 2c, Fig. S1). Data analysis when comparing the individual pre-operative and follow-up samples resulted in defined sets of peptides that were highly individual-specific but similar at both timepoints. The cosine similarity index (CSI) was remarkably high on average (0.79) for paired samples (*n* = 59 comparisons), while CSI was significantly lower on average (0.04) in unpaired samples (Fig. 2d, Fig. S2). These results demonstrated that the seroreactivity against major antibody epitopes remained similar in time in the same subject but differed between subjects.

### BC patients with CPSNP have distinct pre-surgery IgG profiles

**Fig. 3:**
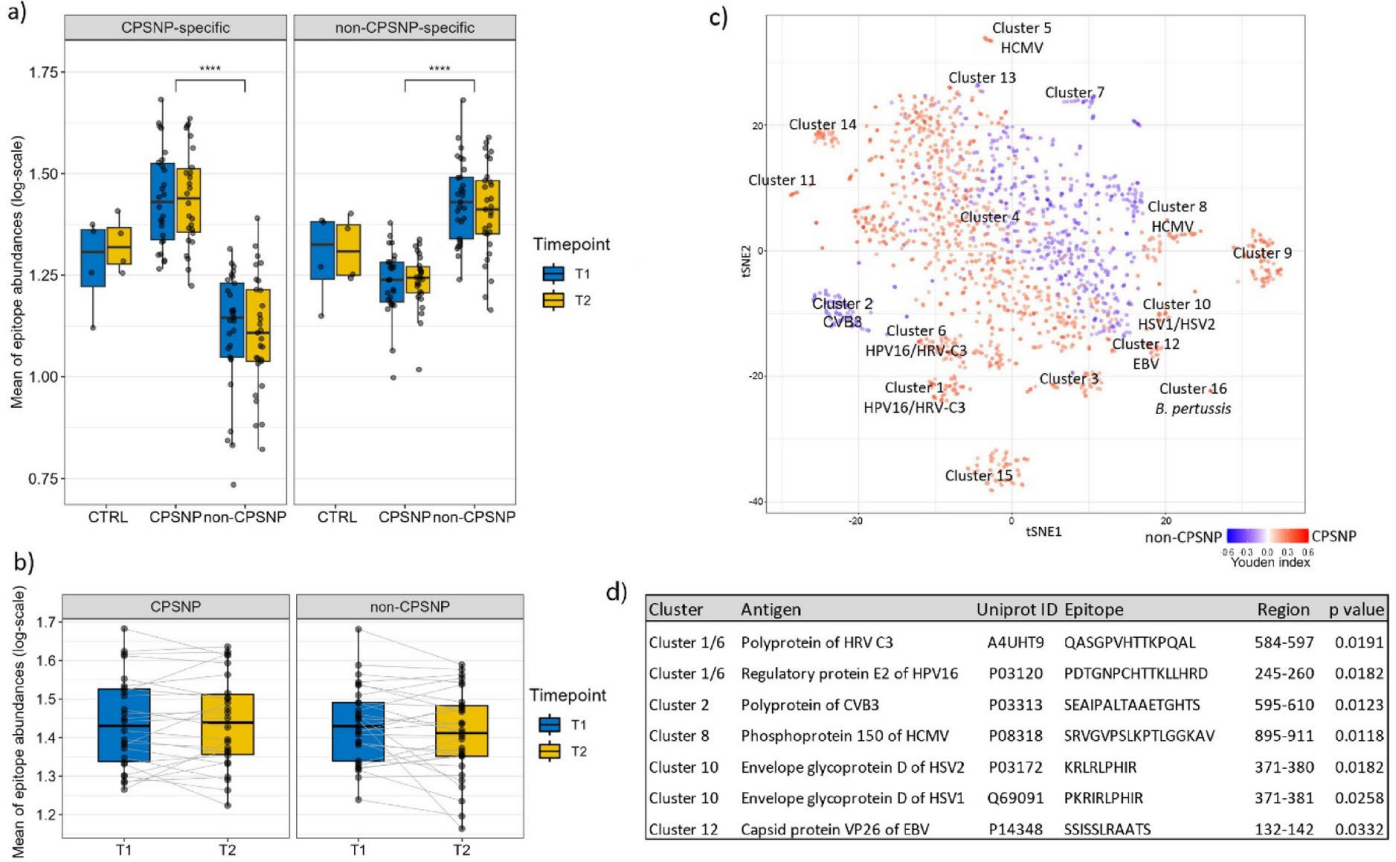
Distinct antibody immune response for CPSNP relates to antigens of common pathogens. (**a**–**c**) Annotation analysis of group discriminative epitope mimics resulted in epitopes (*n* = 1882) showing sequence similarity to common pathogens (*n* = 79) from the Immune Epitope Database (IEDB) (Table S1). 4188 antigenic determinants of common pathogens were selected. Summarized log-transformed values of seroreactivity to these IgG bound epitopes in each sample of CPSNP (*n* = 26), non-CPSNP (*n* = 29) or CTRL (*n* = 4) groups are depicted in a boxplot. Wilcoxon Rank Sum test, p-values adjusted with FDR: **** *p* ≤ 0.0001. (**b**) Pairwise analysis of pre-operative (T1) and follow-up (T2) samples of CPSNP (*n* = 26) and non-CPSNP (*n* = 29) patients. Summarized log-transformed values of seroreactivity to IgG bound epitopes in each sample are depicted in a boxplot. Spearman rho and p-values between timepoint samples showed high correlation: ρ = 0.88, *p* = 1.7*10^− 6^ (CPSNP) and ρ = 0.80, *p* = 1.6*10^− 6^ (non-CPSNP). (**c**) t-SNE analysis of 1882 antibody epitopes identified 17 different clusters (Table S2). For t-SNE and clustering analysis, the abundance of immune response values to 1882 epitopes in 63 cohort pre-operative samples (27 CPSNP, 30 non-CPSNP and 6 CTRL) were normalized to 3 million reads and log-transformed. Epitope clusters were detected using the R package Rtsne (dims 2, perplexity 15, max_iter 500) and R package dbscan (epsilon 2, minPts 10) (Table S2). (**d**) Seven epitopes from different clusters stood out as the most interesting group-discriminating epitopes that aligned to 79 different human pathogen epitopes presented in IEDB (Table S1). The sequences of these epitopes belonging to common human viruses are depicted with amino acid regions. A permutation-based test was performed to evaluate statistical significance of each aligned protein–motif pair (Table S2). For each pair, the protein sequence was held constant while the amino acid positions of the matched motif were randomly shuffled 10,000 times to generate permuted motif variants preserving amino acid composition. Each permuted motif was aligned to the target protein sequence, and the number of exact matches was recorded. The empirical p-value was calculated as: (number of permuted matches + 1)/(number of permutations + 1) and p-values were adjusted with FDR. The highest adjusted p-value is shown. AUC: area under the ROC curve; B. pertussis: *Bordetella pertussis*; CPSNP: chronic post-surgical neuropathic pain; CTRL: control; CVB3: Coxsackievirus B3; EBV: Epstein-Barr virus; CMV: human cytomegalovirus; HRV-C3: human rhinovirus C3; HSV1/2: Herpes simplex virus 1/2; T1: timepoint 1; T2: timepoint 2.

To define differences in antibody epitope profiles among study groups, we performed an unsupervised clustering analysis of the most abundant (immunodominant) peptides detected by MVA and determined group-specific epitopes (Fig. S1). To further investigate the origin of these immune features, we aligned the epitope sequences (*n* = 9344) to the peer-reviewed epitopes from the IEDB with exact matching. This annotation analysis identified 1882 epitopes showing high sequence similarity with the reported epitopes of 79 common human pathogens (Table S2). Table S2 shows annotated linear epitopes associated with pathogen antigens. Antibody immune response to these epitopes was detected in both pre-surgery and follow-up (Timepoints T1 and T2) samples from the patients with CPSNP (Fig. 3a). Pairwise data analysis confirmed the persistence of immune response to the selected epitopes over time (Spearman rho and p-values between timepoint samples showed high correlation: ρ = 0.88, *p* = 1.7*10^− 6^ (CPSNP) and ρ = 0.80, *p* = 1.6*10^− 6^ (non-CPSNP), Fig. 3b). Further clustering analysis resulted in 17 distinct pathogen-associated epitope clusters (Fig. 3c). Annotation analysis indicated that, while most clusters were heterogenous and contained epitopes of different viral antigens (Cluster 4 and 15, for example, Table S2), some clusters were very clearly linked to a specific epitope of a specific pathogen (Fig. 3c). For example, Clusters 5 and 8 were strongly associated with the human cytomegalovirus (CMV) antigen of phosphoprotein 150 (pp150). Clusters 10 and 12 contained epitopes associated with the glycoprotein D (gpD) of human herpes simplex viruses (HSV1 and HSV2). Cluster 2 was more distinct for non-CPSNP than for CPSNP and was enriched in epitopes associated with enteroviruses (Fig. 3c and Table S2). We then quantified the antibody response against these major epitope clusters (Fig. 3c) that were specifically associated with CPSNP. Amongst these, antibody responses against the epitopes of seven viruses emerged as the most discriminating (Tables S2- S3), specifically against antigenic determinants of the polyprotein of human rhinovirus (HRV C3); regulatory protein E2 of human papilloma virus 16 (HPV16); polyprotein of coxsackievirus B3 (CVB3); pp150 of CMV; glycoprotein D of HSV1 and HSV2; capsid protein VP26 of EBV (Fig. 3d). We further hypothesized that some of the identified epitopes related to these common pathogens could be linked to autoimmune mimicry (Table S4). We observed accumulations of immune response targeting extracellular domains of various human protein or secretory protein antigens, as well as those that could directly influence pain signaling. Among others, for example, the epitope of HRV-C3/HPV16 (Cluster 11/Cluster 6) showed mimicry of the epitope of the human collagen alpha-3(VI) chain, a beaded filament collagen shown to have a role in cancer and acute wound healing (UNIPROT ID P12111) (Table S4). These results indicated that a high pathogen load was a dominating feature differentiating patients with or without CPSNP.

### Viral seropositivity by ELISA confirm the MVA data

**Fig. 4:**
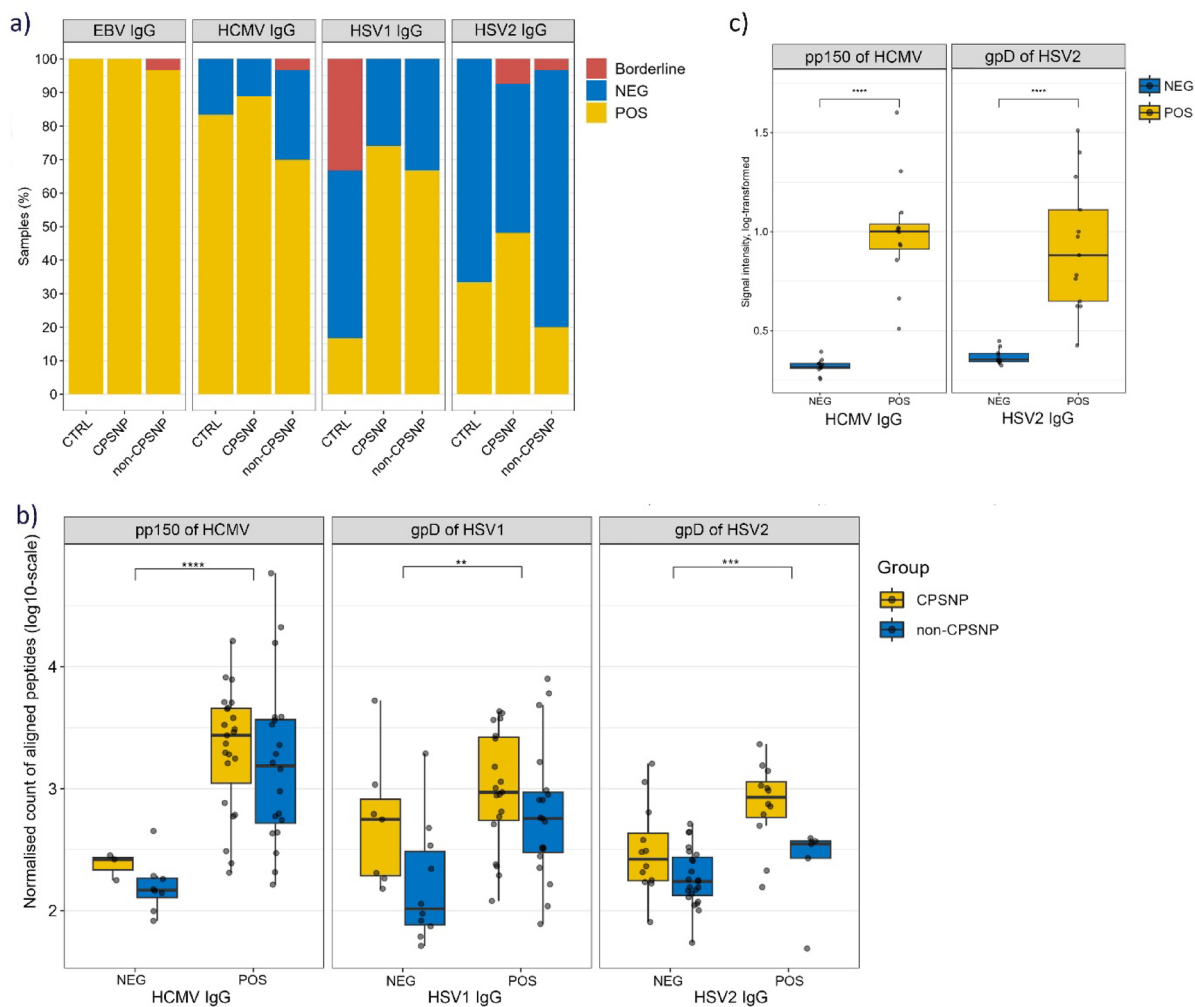
Serology findings of high seropositivity confirm the chronic pathogen burden in CPSNP and non-CPSNP groups. (**a**) IgG response measured by ELISA for common viruses and/or their specific antigens. Serology was measured for EBV (Anti-EBV-CA IgG ELISA, 2791–9601 G), CMV (Anti-CMV, EI 2570–9601 G), HSV1 (Anti-HSV1-gC1, EI2531-9601-2G), HSV2 (Anti-HSV2-gG2, EI2532-9601-2G) for 59 clinical samples ((CPSNP (*n* = 26), non-CPSNP (*n* = 29), CTRL (*n* = 4)). The percentage of HSV seropositivity was higher in the CPSNP group than in non-CPSNP or CTRL groups (Fisher’s Exact Test *p* ≤ 0.05) whereas statistically significant only for HSV2 (Fisher’s Exact Test *p* = 0.027). CPSNP group – 24/3/0 CMV, 27/0/0 EBV, 20/7/0 HSV1, 13/12/2; non-CPSNP – 21/8/1 CMV, 29/0/1 EBV, 20/10/0 HSV1, 6/23/1 HSV2; CTRL – 3/1/0 CMV, 5/1/0 EBV, 1/3/2 HSV1, 2/4/0 HSV2, (numbers referring to POS/NEG/B). (**b**) MVA-detected immunodominant epitopes classify seroresponse to herpesviral antigens pp150 of CMV, gpD of HSV1 and gpD of HSV2. Summarized seroreactivity values of IgG-bound peptides that contained epitopes with sequence similarity to the three epitopes of common herpesviruses are shown as cohort samples are divided by serology status (NEG, POS) and groups (CPSNP, non-CPSNP); Wilcoxon Rank Sum test, p-values adjusted with FDR, ** *p* ≤ 0.01, ***p ≤ 0.001, ****p ≤ 0.0001. (**c**) Epitopes of pp150 of CMV and gpD of HSV1/2 were validated using epitope-specific ELISA with CMV and HSV2 POS/NEG serum samples and peptides displaying epitope of pp150 of CMV (left: *n* = 23) and epitope of gpD of HSV2 (right: *n* = 23). Signal intensities were calculated as Signal/Background ratio (RLU). Wilcoxon Rank Sum test, FDR-adjusted p-values: ****p ≤ 0.0001. CPSNP: chronic post-surgical neuropathic pain; CTRL: control; EBV: Epstein Barr virus (human gammaherpesvirus 4); CMV: human cytomegalovirus (human betaherpesvirus 5); HSV1/2: Herpes simplex virus 1/2 (human herpesvirus 1/2); NEG: seronegative; POS: seropositive; B: borderline RLU: relative light unit.

We analyzed overall seropositivity to EBV, CMV, HSV1 and HSV2 and observed that those for EBV and CMV were prevalent across all study groups, whereas seropositivity for HSV1, and particularly HSV2, were less common (Fig. 4a, Table S1). The percentage of HSV2 seropositive individuals was higher in the CPSNP group than in the non-CPSNP or CTRL groups (HSV2: 48.2%/20.0%/33.3%, Fisher’s Exact test p value 0.027) (Fig. 4a). Next, because the annotation analysis of the antibody profiles to IEDB-reviewed epitopes revealed clear links with common human pathogens, we used the overall seropositivity results to validate the findings of MVA. We assessed the potential of seroreactivity to herpesviral antigens (pp150 of CMV, gpD of HSV1, gpD of HSV2) for accurate classification (Fig. 4b). The antibody response to the examined epitopes was significantly higher in the seropositive samples (Wilcoxon Rank Sum test, FDR-adjusted *p* < 0.01, Fig. 4b). Additionally, the response to gpD antigen of HSV2 was greater in CPSNP samples than in non-CPSNP samples (Wilcoxon Rank Sum *p* < 0.05, Fig. S3). Finally, an epitope-specific ELISA using peptides corresponding to epitopes of pp150 CMV (_892_GRGSRVGVPSLKPTLGGKAV_911_) and gpD HSV2 (_368_MAPKRLRLPHIRDDD_383_) independently confirmed that the MVA findings were genuine targets of the IgG response associated with these pathogens and potentially linked to neuroinflammation (Fig. 4c, Fig. S4).

### Predictive value of immune load to pathogens for CPSNP in breast cancer patients

**Fig. 5:**
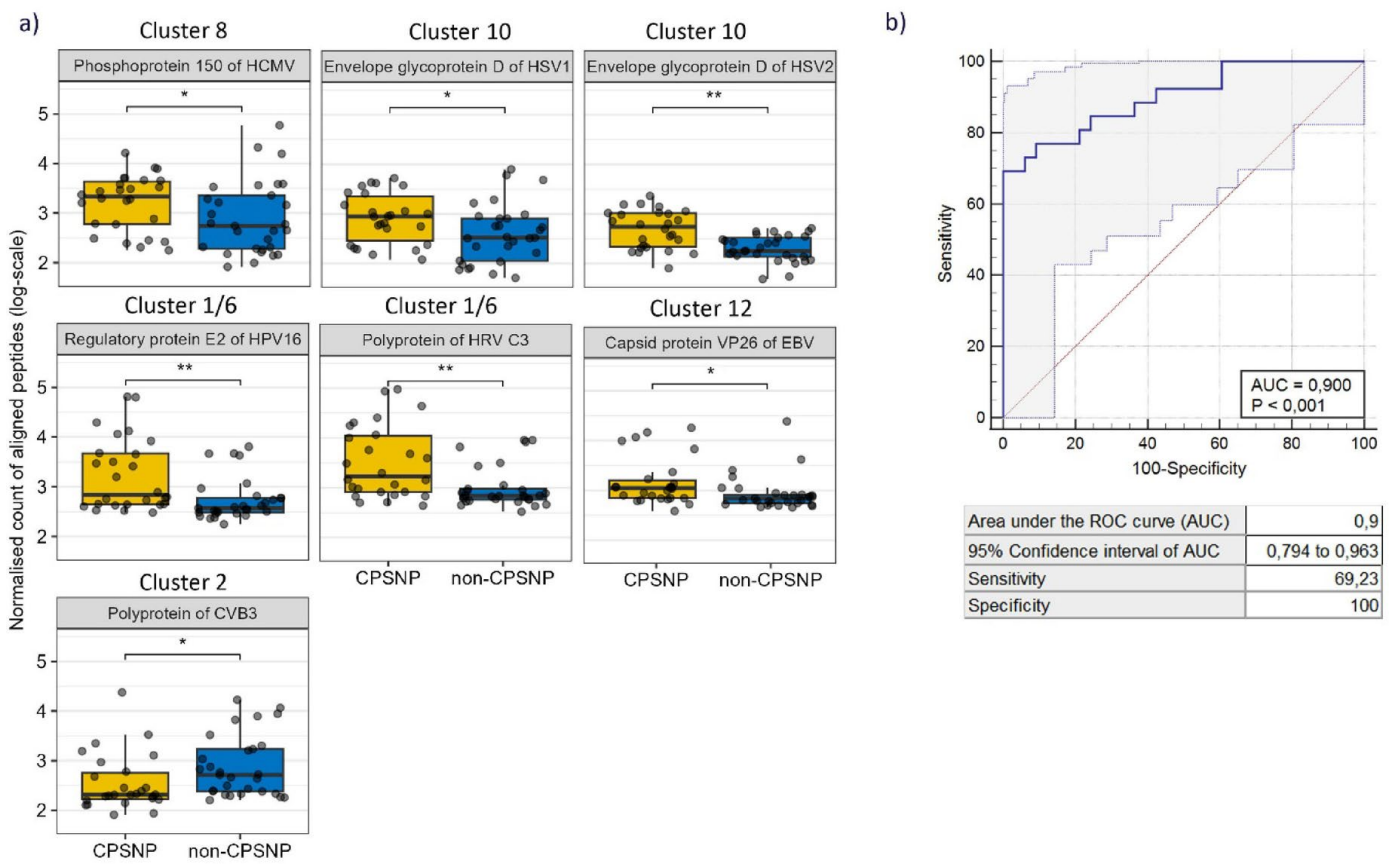
Antibody response to seven highly antigenic epitopes of common viruses as candidate markers for predicting CPSNP pre-operatively. (**a**) Seven epitopes, including those of pp150 of CMV, gpD of HSV1 and HSV2, polyprotein of HRV C3, regulatory protein E2 of HPV16, capsid protein VP26 of EBV, and polyprotein of CVB3, were deemed most discriminating (Tables S2 and S3). Summarized values of IgG-bound peptides with sequence similarity to the seven epitopes from Fig. 3d are depicted in a boxplot for each pre-surgery sample of the CPSNP (*n* = 26) or non-CPSNP (*n* = 29) groups. Wilcoxon Rank Sum test, p-values FDR adjusted: * *p* ≤ 0.05; ** *p* ≤ 0.01. (**b**) Logistic regression model with values of antibody responses to five of these epitopes (pp150 of CMV, gpD of HSV2, E2 of HPV16, VP26 of EBV, polyprotein of CVB3) in pre-operative samples predicts CPSNP with high specificity (1) and sensitivity (0.69) (AUC 0.9). The observed cross-validated AUC was 0.85 and was significantly greater than expected by chance based on a permutation test (*p* = 0.00089). Groups compared: CPSNP (*n* = 26); non-CPSNP + CTRL (*n* = 29 + 4). AUC: area under the ROC curve; CPSNP: chronic post-surgical neuropathic pain; CTRL: control; CVB3: Coxsackievirus B3; EBV: Epstein-Barr virus (human gammaherpesvirus 4); CMV: human cytomegalovirus (human betaherpesvirus 5); HPV16: human papillomavirus 16; HRV-C3: human rhinovirus C3; HSV1/2: Herpes simplex virus 1/2 (human herpesvirus 1/2).

The marked difference in seroreactivity to herpesviruses between the CPSNP and non-CPSNP groups led us to explore the potential of using seroreactivity to antigenic epitopes of the seven common viruses, both against individual viruses and collectively for classification. Individually, antibody responses to single epitopes of CMV, EBV, HRV, HPV16, HSV1 and HSV2 were notably higher in pre-surgery samples of CPSNP than non-CPSNP samples (Fig. 5a). Anti-CVB3 was an exception, as the responses against its epitope were lower in CPSNP than in non-CPSNP samples. In post-operative samples collected 4–9 years after surgery, the seroresponse to most epitopes remained similarly associated with CPSNP (Fig. S5).

We then analyzed all seven features to develop a logistic regression model aimed at predicting CPSNP in BC patients. These data showed that the seroresponse to the defined set of viral epitopes of CVB3, EBV, CMV, HPV-16, and HSV-2, presented a novel predictive biomarker that distinguished CPSNP patients from non-CPSNP patients pre-surgery, achieving a balanced accuracy of 84.6% (Fig. 5b). The observed cross-validated AUC was 0.85 and was significantly greater than expected by chance based on a permutation test (*p* = 0.00089). Baseline clinical characteristics were comparable between the CPSNP and non-CPSNP groups^19^. The variables demonstrating minor difference between the two groups were age and BMI before (T1) and after surgery (T2). The reported biomarker associations remained stable after age and BMI adjustments (Fig. S6).

This model was validated on an independent control cohort^16,17^ comprising age-, sex-, and country-matched individuals (*n* = 28, achieving AUC = 0.806, with a sensitivity of 0.73 and a specificity of 0.75, Fig. S7). Altogether, our findings suggested that immunity load to pathogens may play a significant role in modulating pain signaling and could provide new avenues for therapeutic interventions.

## Discussion

In this study, we present a hypothesis-free analysis of antibody epitopes in patients with painful (CPSNP) and painless (non-CPSNP) nerve injury after BC surgery, using MVA immunoprofiling. MVA uncovered highly individualized immunoprofiles in the paired samples across study groups (pre-operative and follow-up), while also identifying shared immunoreactive features. We identified 1882 epitopes that were differently targeted by antibody response in the two study groups, and these epitopes showed high sequence similarity to antigens of common human pathogens. These included epitope mimics of the polyprotein of HRV-C3, regulatory protein E2 of HPV16, phosphoprotein 150 of CMV, capsid protein VP26 of EBV and envelope glycoprotein D of HSV1 and HSV2, whereas epitopes of polyprotein of CVB3 were associated with painless nerve injury. Similar immune responses to the epitopes of these common human viruses were detected in the samples collected before surgery and after the follow-up showing that the findings persisted over time. Five of these specific epitopes (epitopes of CMV, EBV, HSV2, CVB3, and HPV-16) could be potentially clinically useful as preoperative predictors of CPSNP in patients undergoing BC surgery (AUC of 0.9, 95% CI, 0.794–0.963). This suggests that immune load linked to common viruses is associated with an increased risk of developing CPSNP.

The antibody repertoire reflects both the past and current activity of the adaptive immune system. Immune response against previously encountered environmental pathogens has been identified as a potential risk factor for various autoimmune disorders^20^, as well as in other contexts, including psychiatric disorders^21^. Recently, serological evidence of multiple infections was associated with multisite chronic pain^22^. To the best of our knowledge, this is the first study to report an association between antibody responses against common and latent viruses and the risk of developing persistent pain after surgical nerve injury.

Neuropathic pain (NP) is caused by disease or a lesion in the somatosensory system. However, not all nerve lesions become chronically painful. Immune system has a major contribution in the resolution on pain after nerve injury but also in the maladaptive mechanisms that lead to persistent pain^23,24^. Moreover, cellular immune response to nerve injury is regarded as a possible therapeutic target^25^. The crosstalk between immune system and NP includes interactions with both innate and adaptive immunity. This process involves almost every immune system component and cell type, including macrophages, neutrophils, T cells, glial cells, cytokines, chemokines and inflammatory mediators^7,13,23–25^. However, the role of humoral immune mechanisms has been less studied in the context of painful nerve injury, although growing evidence implicates its potential contribution in certain persistent pain conditions^9,10,26,27^.

Antibodies may exert pronociceptive effects through immune-complex formation, complement activation, and Fc-receptor–mediated sensitization of peripheral nociceptors, providing a potential mechanistic link between adaptive immunity and pain processing^9,10^. For example, IgG from complex regional pain syndrome (CRPS) and fibromyalgia patients can transfer pain-like hypersensitivity to rodents^26,27^. Furthermore, in a subset of fibromyalgia patients, autoantibodies bind to satellite glia cells in the dorsal root ganglia (DGR) of the sensory nerves^28^. In the context of nerve injury, a mouse model showed accumulation of IgG in DRG and dorsal spinal cord with potential pro-nociceptive effect^13^. A clinical study of spinal cord injury patients showed an antibody-mediated autoimmune response that was associated with a higher need of NP medication^12^. However, there is a lack of clinical studies concerning the role of antibodies in painful peripheral nerve injury.

Our data suggest an elevated immune response to multiple human herpesviruses as a risk factor for developing persistent NP after breast cancer surgery. Human herpesvirus infections or their post-infection syndromes have been associated with persistent NP in different contexts, with post-herpetic neuralgia the most well-known. Apart from these, the association of latent herpesvirus infections and other NP conditions is less studied. A rare unilateral pain syndrome with NP features has been reported in association with recurrent HSV infections^29^. Indeed, HSV1 and − 2 remain latent in the ganglia of sensory nerves with the potential of clinical or subclinical reactivation later in life. Although these viruses are known to reside primarily in trigeminal (HSV1) and sacral (HSV2) ganglia, HSV1 can sometimes reside in thoracic ganglia^30^ suggesting a plausible anatomical link to the CPSNP-related findings in the present study.

EBV infects mainly B cells and epithelial cells, CMV infects monocytes, lymphocytes and epithelial cells. After primary infection the reactivations of CMV and EBV are usually asymptomatic. Previously, seropositivity to EBV has been associated with chronic multisite musculoskeletal pain and inflammatory joint pain^22,31^. Other chronic pains are common comorbidities in NP, and they associate with risk of developing CPSNP after surgery^32,33^. High antibody titers to both EBV and CMV have been associated with elevated levels of other inflammatory mediators such as CRP and IL-6 indicating that the activity of multiple herpesviruses can drive general inflammation^34^. Furthermore, general inflammation and higher levels of pro-inflammatory cytokines have been associated with NP in different contexts in both pre-clinical and clinical studies^6,7,23^. This is in line with our data showing elevated antibody response to multiple herpesviruses in CPSNP. High antibody levels to latent herpesviruses are indirect indicators of viral reactivations and they also serve as an indicator of immune dysregulation, due to their associations with elevated levels of inflammation^34,35^. The compromised immune function which predisposes individuals to recurrent and prolonged herpesviral reactivation specifically could be the underlying factor for the elevated risk of CPSNP following nerve injury^36^. However, our study does not provide direct information about immune function, inflammation, or herpesvirus reactivations and further studies are needed to confirm this hypothesis.

Notably, compared with the CPSNP group, the non-CPSNP group patients exhibited an elevated IgG response targeting epitopes that mimic the polyprotein of CVB3, a common human enterovirus causing flu-like symptoms and sometimes myocarditis. It remains unclear how antibody response to CBV3 could associate with favorable outcome in terms of pain development after surgical nerve injury. The clinical relevance of this finding needs further evidence. Interestingly, in a recent MVA study, robust antibody response to a conserved enteroviral epitope was associated with better cardiovascular health^17^.

Our modelling results suggest that antibody response to specific antigen epitopes of CMV, EBV, HSV2, CVB3, and HPV-16 prior to surgery could be useful predictors of CPSNP in patients undergoing BC surgery. There are several mechanisms that could be hypothesized. Firstly, antibodies could be nociceptive per se, like in CRPS and fibromyalgia. Secondly, antibodies may jointly induce general inflammation^34^, which interferes with normal nerve regeneration and leads to pain sensitization^24,36^. Thirdly, antibodies may be markers of compromised immune functions, which would be the biological cause of lack of regeneration and pain. Lastly, reverse causality is also worthy of consideration, because chronic NP is associated with increased stress and psychological burden^32,37^ and stress is known to induce reactivations of herpesviruses. All in all, our findings support the hypothesis that prior infections and viral re-activations potentially prime neuroimmune pathways that facilitate the development and persistence of NP following surgical injury. Since our study does not provide direct information about the underlying mechanisms, further research is necessary to clarify the role of antibody responses in NP development.

Strengths of this study include a prospective setting of thoroughly characterized study groups of painful and painless nerve injury patients after BC surgery. Our data is unique, because a similar setting is very difficult to obtain in most other NP etiologies where the onset of nerve lesion and NP are usually unpredictable. Another strength of this study is its comprehensive immune response analysis. The interaction between the immune system, inflammation, and pain is intricate. MVA allowed us to screen a vast set of peptides, accelerating the discovery process of antigens associated with chronic pain at the epitope level. However, our study does not provide direct information on active infection, pathogen load, or viral reactivation. The patient cohort is small, but the patient groups were carefully characterized with a detailed assessment of NP along with preoperative and follow-up samples with timespan up to nine years. These together add considerable depth to our study. Our results may not be directly generalizable to men and to other NP etiologies, because our study groups comprised of BC treated women with surgical nerve injury.

Our analysis demonstrated that the antibody response profiles in BC patients experiencing CPSNP showed reactivity to multiple viral antigens. These immune response patterns may be candidates as predictive markers assessing the risk of pain development during the preoperative stage. This finding could pave ways to new therapeutic opportunities, such as use of antiviral drugs, for the prevention and management of neuropathic pain. Since our results from clinical patients are preliminary, we encourage further studies to elucidate the multidisciplinary mechanisms and potential treatment avenues in persistent NP.

## Patients and methods

### Patient selection

We have previously described the diagnosis of NP in 251 women whose treatment for BC involved axillary surgery and ICBN resection (as reported by the surgeons). We examined the patients for the presence or absence of CPSNP of the ICBN 4–9 years after surgery^32^. All patients were recruited from a previous prospective cohort of 1000 women operated on for BC at the Helsinki University Hospital during 2006–2010^38^. Thus, we were able to use data and samples collected preoperatively from the patients in the current study. During surgery, the operating surgeon reported the handling of ICBN (totally resected, partially resected, spared or not identified). Patients with total or partial ICBN resection were invited for the follow-up research visits for the current study. Follow-up research visits took place during 2014–2016, 4–9 years after surgery^32^. The visits included thorough clinical sensory examination by a neurologist (HH) for diagnosis of NP according to stepwise grading criteria (unlikely, possible, probable, definite NP)^39^, and collection of blood samples. The contents of sensory examination and NP grading have previously been reported in detail elsewhere^32^. To fulfill the criteria for definite NP, the pain and sensory alterations had to be located within the area of ICBN innervation and the ICBN resection had to be confirmed by the operating surgeon. Because of the surgeon’s report of nerve lesion, no additional diagnostic tests are needed to fulfill the criteria of definite NP^39^. We used the “worst pain past week” in the Brief Pain Inventory (BPI) long form to measure pain intensity on a Numerical Rating Scale (NRS, 0–10). NRS ≥ 4/10 was considered to reflect at least moderate pain intensity and clinically meaningful pain^40^. All patients were asked for the presence of pain in other body locations (back, neck, joints, head, other) and use of current pain medications. In addition, Douleur Neuropathique 4 (DN4) questionnaire^41^ was completed for each patient with pain.

For the current study assessing the role of antibody response profiles in CPSNP, we selected subgroups using the following criteria: (1) the CPSNP group included patients fulfilling the criteria of definite NP^39^ and pain intensity ≥ 4/10 on the NRS and (2) the non-CPSNP group included patients with ICBN resection with no CPSNP and no other pains. Patients with ongoing cancer treatment, neurological or autoimmune diseases were excluded from the present analysis. Patient selection is presented in Fig. 1. For technical control purposes, preoperative and follow-up samples from six BC-operated patients without ICBN injury and without pain were included in the MVA studies. These patients were from the same original cohort as the analyzed groups^32^.

The Coordinating Ethics Board of the Helsinki and Uusimaa Hospital District approved the study (149/13/03/00/14) which was also registered in ClinicalTrials.gov (NCT02487524). This study was performed in accordance with the Declaration of Helsinki. All patients gave written informed consent.

### Breast cancer treatments

The cancer treatment data were accessible from the previous study^38^. All patients had been operated on for unilateral BC, and none had received neoadjuvant treatment. Breast surgery was either mastectomy or breast conserving surgery (BCS) accompanied with axillary surgery, which was either sentinel lymph node biopsy (SLNB) or axillary lymph node dissection (ALND). The resection of the ICBN (either total or partial) was verified by the operating surgeon. Oncological treatments, including radiotherapy, chemotherapy, and endocrine treatment, were administered according to national treatment protocols. The chemotherapy regimen comprised a combination of docetaxel and CEF (cyclophosphamide, epirubicine, and 5-fluorouracil). Trastuzumab was administered to the patients with HER2 (human epidermal growth factor 2) -positive BC. Tamoxifen (premenopausal women) or aromatase inhibitor (postmenopausal women) comprised the endocrine treatment.

### Sample collection

Plasma samples for the MVA were collected at two separate timepoints: at induction of anesthesia for the BC surgery (Timepoint 1 [T1]) and at the research visit 4–9 years after surgery (Timepoint 2 [T2]). Plasma from blood samples collected into ethylenediaminetetra-acetic acid (EDTA) tubes was extracted by centrifugation at 3000 revolutions per minute (RPM) for 10 min, and transferred to cryogenic vials, which were immediately frozen and stored at −80 °C. Both the preinjury and follow-up samples were drawn and prepared by the same research nurse. The follow-up research visit protocol included the analysis of basic laboratory parameters and inflammatory markers, including high-sensitivity C-reactive protein (hs-CRP). The collection of study samples has been reported previously^32^.

### Mimotope variation analysis

Sample (*n* = 126) processing by MVA was performed according to standard MVA protocols^14^. In brief, plasma samples (*n* = 126, 2 µl of plasma per analysis) were incubated with *Escherichia coli* M13 phage library displaying random 12-mer peptides (complexity 1 × 10^9^ peptides; NEB, E8111L). IgG–phage complexes were isolated with protein G-coated magnetic beads (NEB, S1430) and amplified by Phusion High Fidelity PCR (Thermo Scientific) for next-generation sequencing. An average of 3 million peptide-encoding DNA sequences per sample was achieved: 5000 most abundantly detected peptides by abundance values (read counts) from each sample were taken into immunoprofile-similarity analysis. For comparisons, the normalized scalar products of peptide count vectors were calculated for the cosine similarity index (R package lsa) that ultimately resulted in a 126 × 126 sample immunoprofile-similarity matrix. Due to not meeting technical criteria, four samples (two controls, one CPSNP and one non-CPSNP), were excluded from downstream group comparison and regression analyses (Table S1).

### Delineating cohort specific epitope responses

#### Cohort-specific epitope selection

To delineate cohort-specific antibody response, the data comparison of the most immunodominant epitopes between CPSNP and non-CPSNP groups was carried out (see schematic overview of the data analysis workflow, Fig. S1). The most immunodominant epitopes were determined by clustering analysis (defining 5–11 positions-long epitopes that all contained five fixed amino acids) and hypergeometric test analysis where SPEXS2 Software was used^14^. Samples of these two groups at either timepoint (T1, before surgery; T2, at the 4–9 years follow-up) were compared with each other using optimal cut-point analysis (R package cutpointr, maximizing the Youden metric) and Student’s *t*-test (R Stats Package, log-transformed). Based on the group comparisons, 9344 epitopes were chosen for further analysis (*t*-test *p* < 0.05 and Youden > 0.25).

#### Alignment analysis on infectious epitopes of IEDB and marker epitope selection

To look for potential mimicry with human and pathogen antigens, group-specific (*n* = 9344) epitopes were exactly aligned with sequences of T and B cell peer-reviewed epitopes in a public database (Immune Epitope Database [IEDB], version: epitope_full_v3.tsv, 2,225,965 epitopes of which 2,214,634 were linear, date accessed: 15.04.2024) (see schematic overview of data analysis workflow, Fig. S1). Based on the relevance (determined by assessed probability of pathogen exposure) and alignment scores, the most relevant microorganisms were selected (*n* = 79, Table S2). To evaluate the statistical significance of specific epitope–motif alignments, we performed permutation-based tests for each aligned epitope–motif pair (*n* = 6254). For each pair, the epitope sequence was held constant while the amino acid positions of the matched motif were randomly shuffled 10,000 times to generate permuted motif variants preserving amino acid composition. Each permuted motif was aligned to the original epitope sequence, and the number of exact matches was recorded. The empirical p-value was calculated as: (number of permuted matches + 1)/(number of permutations + 1) and p-values were adjusted with FDR. 1882 epitopes that showed sequence similarity with the revised epitopes from IEDB, were visualized and clustered using R packages Rtsne (where t-SNE is stochastic neighbor embedding)^42–44^ and dbscan^45^. For clustering analysis, the relative values of immune response to the 1882 epitopes in T1 samples (*n* = 63) were log-transformed. Epitope clusters were identified using the Rtsne function from R’s Rtsne package (dims = 2, perplexity = 15, max_iter = 500) and the dbscan function from the dbscan package (epsilon = 2, minPts = 10). Altogether, 17 different clusters were identified (Table S2). Further, the most relevant epitopes and antigens identified by the study cohort were described by alignment scores (Table S3) where the seven most interesting leads (with the highest alignment scores) were selected (see schematic overview of data analysis workflow, Fig. S1). The total immune response value for each epitope was calculated by summarizing the counts of all unique peptides containing the aligned motifs and log-transformed per sample (Wilcoxon Rank Sum test, p-values unadjusted). Following regression and receiver operating characteristic (ROC) analyses, corresponding visualizations were created using MedCalc Statistical Software (version 17.0.4, MedCalc Software Bvba, Belgium) (see schematic overview of data analysis workflow, Fig. S1). For independent validation (regression and ROC analyses), previously published immunoprofiles were used for country- (Finland), age- (52.9 ± 6.21 years) and sex- (female) matched clinical samples (*n* = 26) with no diagnoses of BC and NP pain^16^.

#### Alignment analysis on autoantigenic epitopes of IEDB

Autoimmune-associated epitopes were filtered from the original IEDB database (version: epitope_full_v3.tsv, export on 15.04.2024) alignment with 1882 queried epitopes (see schematic overview of data analysis workflow, Fig. S1). All autoimmune-associated peer-reviewed B cell epitopes containing 82 infection-related epitope mimics were included in the study (Table S4). For the antigens identified, cellular locations were gained from the UNIPROT database (column intracellular/extracellular, column features – cytoplasm, endoplasmic reticulum, cell membrane, secreted, cell junction, cell projection, chromosome, Golgi apparatus, lysosome, mitochondrion, nucleus, recycling endosome, NA – data not available) (Table S4).

### Epitope validation

For epitope validation, in-house epitope-specific enzyme-linked immunosorbent assay (ELISA) was used to analyze randomly selected samples with proportional seronegative and -positive sample representation (human cytomegalovirus [CMV] pp150, *n* = 23; herpes simples virus 2 [HSV2] gpD, *n* = 23). Peptides containing target epitopes identified by MVA (for CMV pp150 _901_SLKPTLGGK_910_, for HSV2 gpD _370_PKRLRLPHIRDD_383_) and control epitope (_1_AASAASAA_8_) were synthesized with C-terminal addition (GGGDYKDDDKK(biotin), CASLO ApS). Nunc Immobilizer Streptavidin-coated 96-well plates (Thermo Scientific, 436015) were coated with 0.03 µg/ml (3 ng/well) peptides in 1xPBS for 1 h at room temperature and washed with 0.1% PBS-Tween. After blocking unspecific binding with 5% BSA for 1 h and washing with 0.1% PBS-Tween, diluted plasma samples (plasma dilution 1:1000) in 1% BSA 0.1% PBS-Tween were allowed to bind to peptides overnight at + 4 °C. Next day, the plate was washed with 0.1% PBS-Tween and incubated with secondary antibodies (polyclonal anti-human-HRP (1:1000; goat anti-human IgG (H + L) (Invitrogen, 31410) for 1 h at room temperature. Finally, the plate was washed with 1x phosphate-buffered saline (PBS), and 20x dilution of chemiluminescent substrate (SuperSignal™ ELISA Femto Substrate (Thermo Scientific, 37075)) was added. Results were measured within 5 min using a multimodal microplate reader (Hidex Oy). The signal intensities were calculated as Signal/Background ratio (relative light unit [RLU]) (Wilcoxon Rank Sum test p-values, Pearson correlation coefficients).

### EBV, CMV, HSV1 and HSV2 seropositivity

Commercial ELISA tests were used to determine the serology of 59 samples (CTRL [control, *n* = 4], CPSNP [*n* = 26], non-CPSNP [*n* = 29]) to herpesviruses Ebstein-Barr virus (EBV; Anti-EBV-CA IgG ELISA, 2791–9601 G), CMV (Anti-CMV, EI 2570–9601 G), HSV1 (Anti-HSV1-gG1, EI2531-9601-2G), HSV2 (Anti-HSV2-gG2, EI2532-9601-2G) by analyzing samples from T1 (Table S1). Analyses were carried out in accordance with the manufacturers’ specifications. Absorbency was measured at 450 nm multimodal microplate reader (Hidex Oy).

### Statistics

Statistical analyses were conducted with R statistical programming language (v.4.2.3) and RStudio environment (v. 2024.09.1 Build 394). Data were analyzed, graphs were produced and visualized using R packages tidyverse^46^, ggpubr^47^, ggsci^48^ and scales^49^. Cosine similarity indices (CSIs) for sample comparisons based on the top 5000 peptide abundance values and composition were calculated with the cosine function in R package lsa^50^. Boxplots were generated using the style of Tukey with R packages ggpubr and ggplot2^51^. In figures, the upper, middle and lower boxplot lines represent the 75th, 50th, and 25th percentiles, while whiskers represent the largest or smallest value within 1.5x interquartile range above the 75th percentile or below the 25th percentile, respectively. The p-values of Wilcoxon Rank Sum tests were visualized with R ggpubr or ggplot2 packages, and statistical significance is shown where applicable. Student’s *t*-tests (base R Stats Package), along with each epitope’s Youden index calculation analysis (R package cutpointr^52^, maximizing the Youden metric), were used to identify 9344 unique group-differentiating features. MedCalc^®^ Statistical Software (v.20.121.2, www.medcalc.org; 2022) was used to conduct logistic regression and ROC analyses of five pathogen epitopes were used as combinational tests. The proportion of seropositive and -negative samples was evaluated with Fisher’s Exact Test from the R Stats Package^53^. Spearman’s ρ statistic for epitope-specific ELISA and MVA data correlation were calculated with cor.stat from the R Stats Package. Age adjusted logistic regression, ROC/AUC and cross-validation calculations were performed in R with glm() function from “stats”, “pROC”^54^ and “caret”^55^ packages. Model performance was assessed using 5-fold cross-validation. Statistical significance was evaluated using a permutation test in which outcome labels were randomly shuffled 10,000 times, generating a null distribution of cross-validated AUC values. The permutation p-value was calculated as the proportion of permuted AUCs greater than or equal to the observed AUC obtained from the unpermuted data.

## Supplementary Information

Below is the link to the electronic supplementary material.

Supplementary Material 1

Supplementary Material 2

## Data Availability

All data are available upon reasonable request to the corresponding author (clinical data) and Kaia Palm (MVA-data, [kaia@protobios.com](mailto: kaia@protobios.com)).
