# Supplementary material for: Comprehensive antigen profiling predicts post-surgical neuropathic pain in women treated for breast cancer
Source: Sci Rep. 2026 Mar 3;16:12511. doi: 10.1038/s41598-026-41637-6 (PMC13086930; doi:10.1038/s41598-026-41637-6)
Supplement: Supplementary file 2 — Supplementary Material 2 [file 41598_2026_41637_MOESM2_ESM.docx]

**SUPPLEMENTARY MATERIAL**


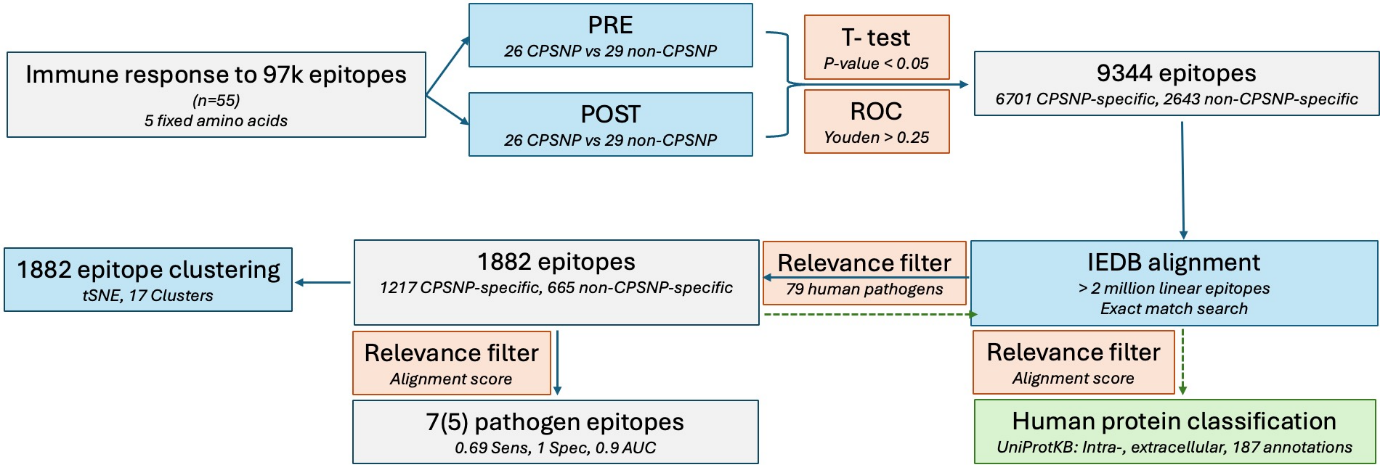
***Figure S1. Schematic overview of the data analysis workflow.*** *To find cohort specific epitope responses, the immune response to the most immunodominant epitopes (n=97 046) was compared between CPSNP (n=26) and non-CPSNP (29) groups (altogether 55 patients). Based on the group comparisons, 9344 epitopes were chosen for further analysis (2643 non-CPSNP-specific and 6701 CPSNP-specific epitopes, for both pre-operative and follow-up sample comparisons: t-test p-value < 0.05 and Youden > 0.25). To look for potential mimicry with human and pathogen antigens, 9344 epitopes were exactly aligned to Immune Epitope Database (IEDB) of full T and B cell peer-reviewed epitopes (epitope_full_v3.tsv, 2,225,965 epitopes of which 2,214,634 were linear epitopes, date accessed: 15.04.2024). 1882 epitopes (1217 CPSNP-specific and 665 non-CPSNP-specific epitopes; Wilcoxon Rank Sum test p <0.05, p-values unadjusted) that showed sequence similarity with the revised epitopes from IEDB and belonged to 79 human pathogens, were visualized and clustered using R packages “Rtsne” and “dbscan” (17 clusters) (****Table S1****). Further, the most relevant epitopes and antigens recognized by the study cohort were described by alignment scores where 7 most interesting leads (with the highest alignment score) were selected (****Table S2****) and 5 of them showed best predictive model for CPSNP (marked as 7(5) on the figure). Autoimmune-associated epitopes were filtered from the original IEDB database alignment with 1882 queried epitopes (****Table S3****). For the antigens recognized in the study, cellular locations were queried from UNIPROT database (column intracellular/extracellular, column features – cytoplasm, endoplasmic reticulum, cell membrane, secreted, cell junction, cell projection, chromosome, Golgi apparatus, lysosome, mitochondrion, nucleus, recycling endosome, NA – data not available).*

 
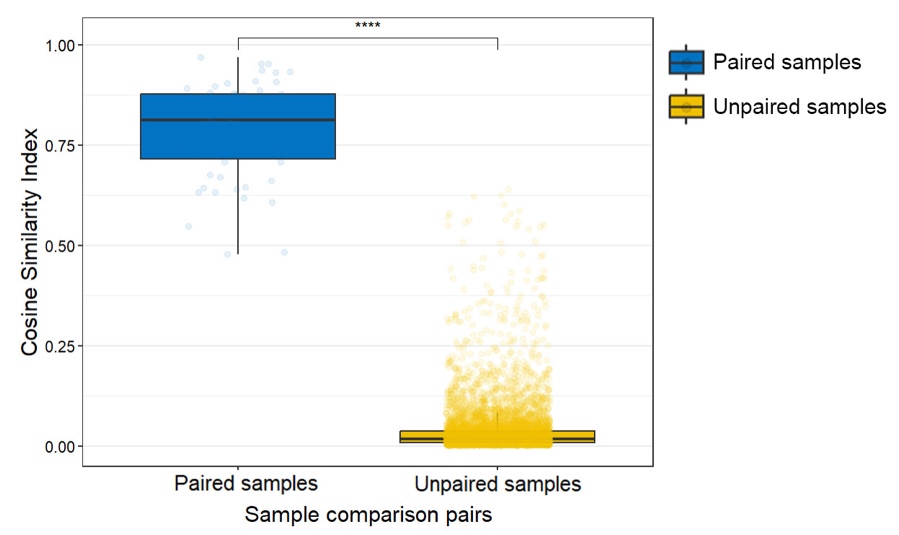


***Figure S2. The cosine similarity index (CSI) was remarkably high (0.79) on average for paired samples (n=59 comparisons), while CSI was significantly lower on average (0.04) in unpaired samples.*** *The 5000 most IgG-bound (abundant) peptide values (read counts) from each sample were taken into immunoprofile similarity analysis. For all sample pairs, the normalized scalar products of peptide count vectors were calculated for the cosine similarity index (CSI, R package “lsa”) and CSI values are depicted. CSI threshold failed in four removed samples (****Table S1****) and these are not depicted. Wilcoxon Rank Sum test, **** p-value 5 *10^-40^.*


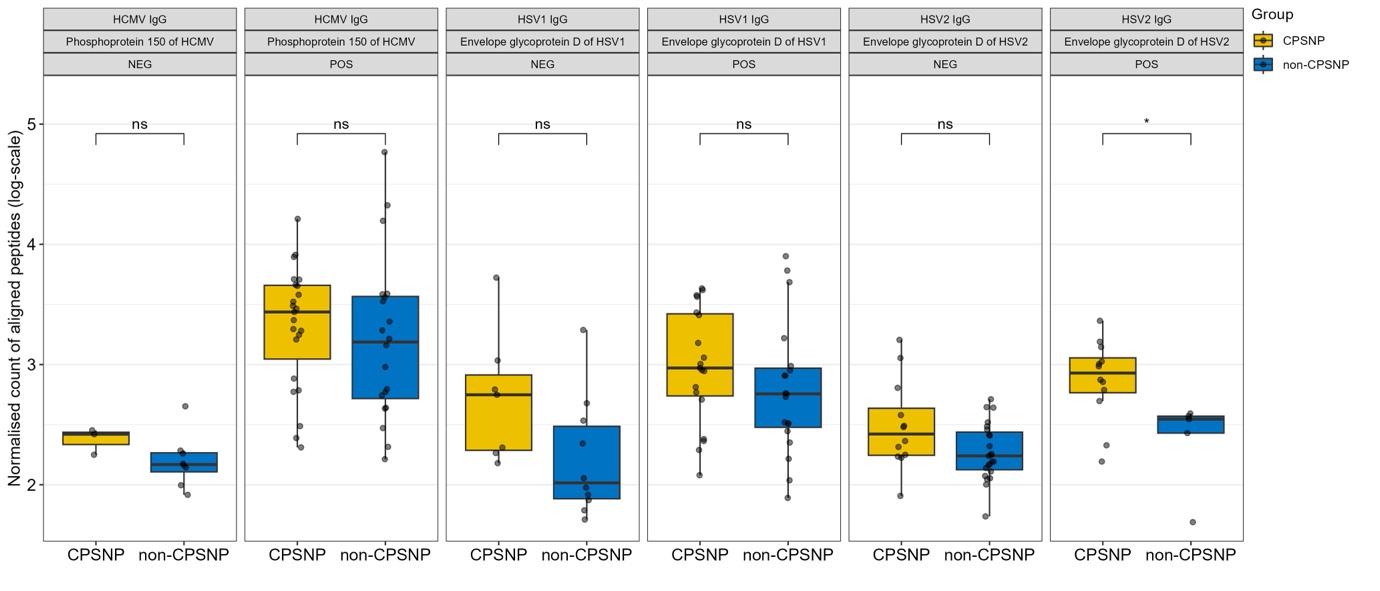
***Figure S3. Seroresponse to epitopes of the pp150 of HCMV, gpD of HSV1 and gpD of HSV2 antigens in CPSNP (n=26) and non-CPSNP (n=29) from MVA data.*** *CMV* *negative n=11, CMV positive n=43, CMV Borderline n=1, (not depicted); HSV1 negative n=17, HSV1 positive n=38; HSV2 negative n=35, HSV2 positive n=17, HSV2 Borderline, n=3 (not depicted). HCMV – Human cytomegalovirus (Human betaherpesvirus 5); HSV1/2 – Herpes simplex virus 1/2 (Human herpesvirus 1/2); NEG – seronegative samples, POS – seropositive samples. Wilcoxon Rank Sum test, p-values unadjusted: * p≤ 0.05*.


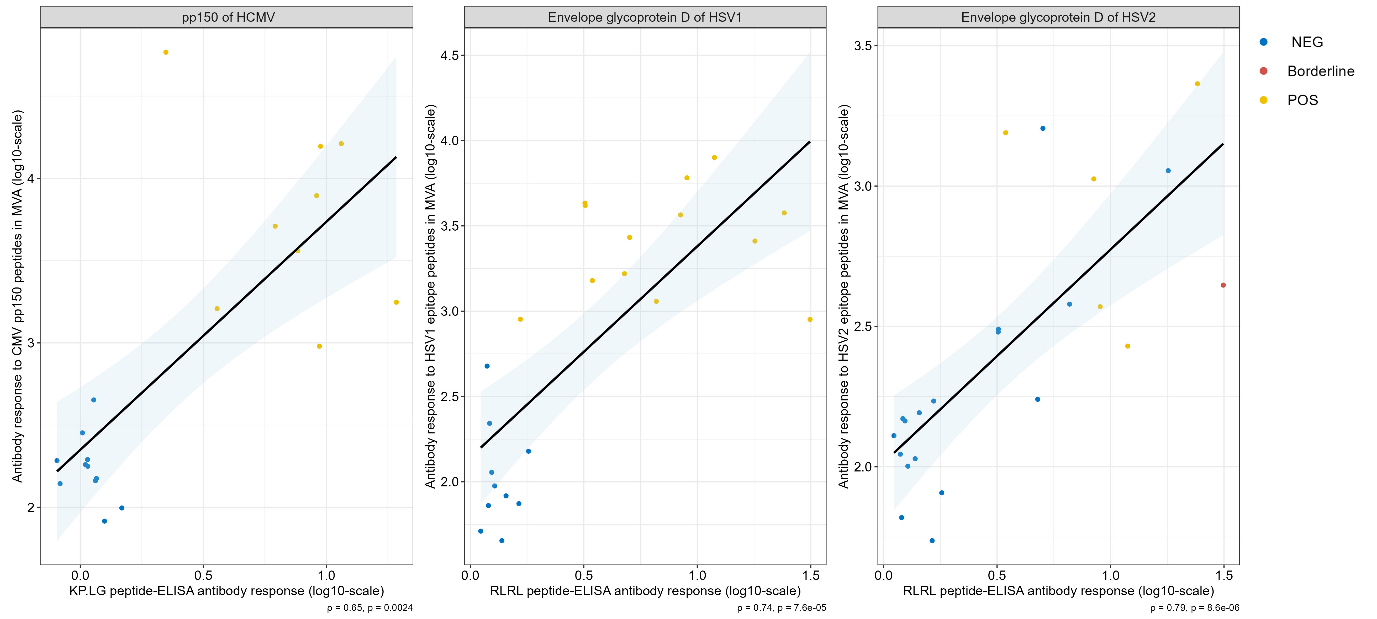


***Figure S4. Comparison of MVA and epitope-specific ELISA.*** *Spearman correlation analysis of seropositivity measurements of pp150 of HCMV, gpD of HSV1 and gpD HSV2 with MVA and epitope-specific ELISA. Samples were chosen randomly from the study cohort to have a balanced serological representation. Sample sizes: pp150 of HCMV (n=20, 3 samples (10021, 10245, 10955), which were removed from data analysis are not depicted on the panel); Envelope glycoprotein D of HSV1 and HSV2 (n=23). HCMV ρ=2.3e-03, P=2.4e-03; HSV1 ρ=7.6e-05, P=1.4e-04; HSV1 ρ=8.6e-06, P=2.58e-05. y-axes - MVA seropositivity values (normalized count of aligned peptides (log-scale); x-axes - epitope-specific ELISA seropositivity values (the signal intensities were calculated as Signal/Background ratio (relative light unit - RLU)). ρ - Spearman's rank correlation coefficient.P-values corrected with FDR.Colour code refers to serological measurements with commercial anti-HCMV, anti-HSV1 or anti-HSV2.*

 
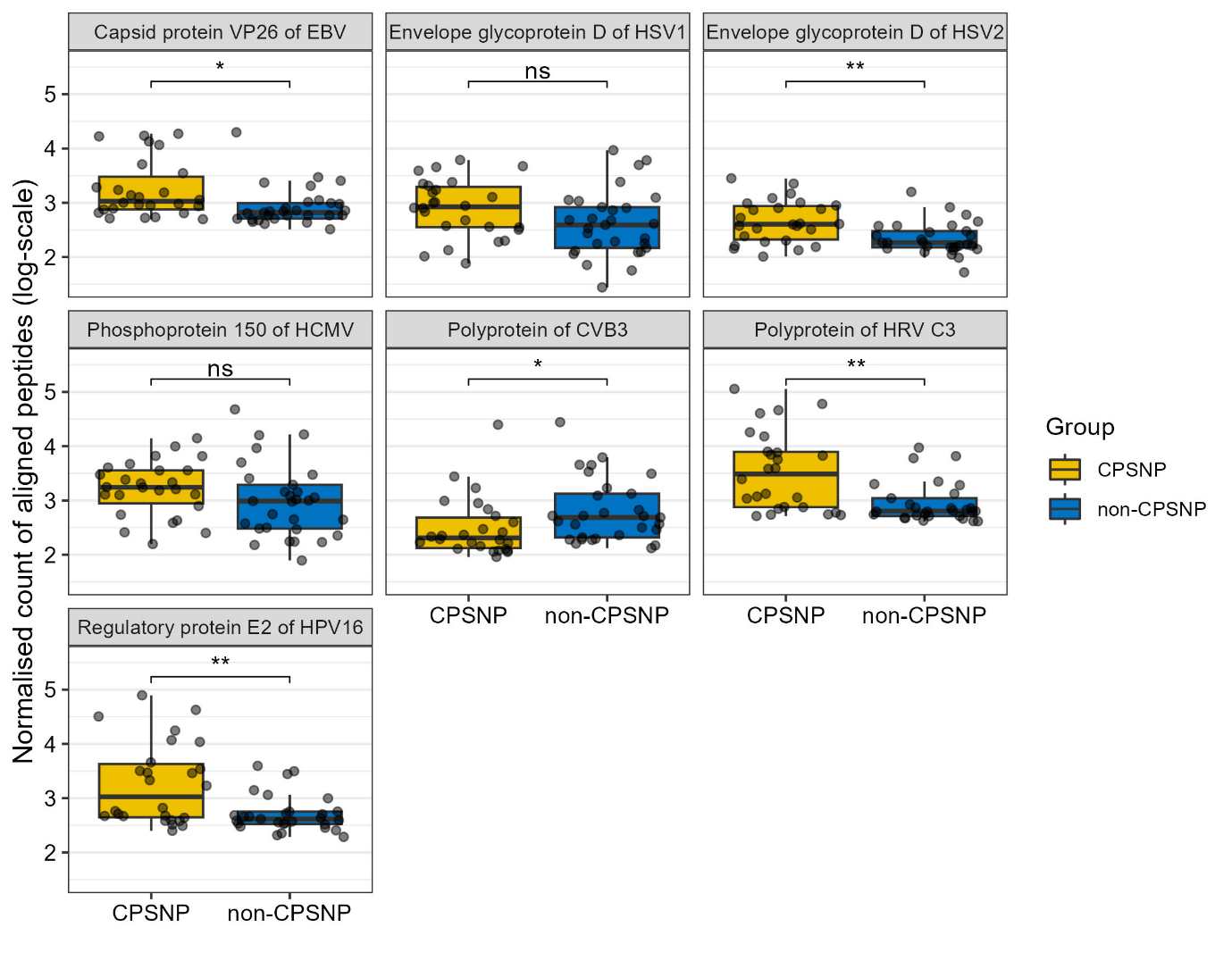


***Figure S5. Highly antigenic epitopes of common pathogens are associated with CPSNP.****Post-surgery samples from CPSNP(n=26)/non-CPSNP(n=29). HCMV – Human cytomegalovirus (Human betaherpesvirus 5); EBV – Epstein Barr virus (human gammaherpesvirus 4); HRV-C3 – Human rhinovirus C3; HSV1/2 – Herpes simplex virus 1/2 (Human herpesvirus 1/2); CVC3 - Coxsackievirus B3; HPV16 - Human papillomavirus 16; CPSNP - chronic post-surgical neuropathic pain. Wilcoxon Rank Sum test, p-values adjusted with FDR: ns p> 0.05, * p≤ 0.05, ** p≤ 0.01.*


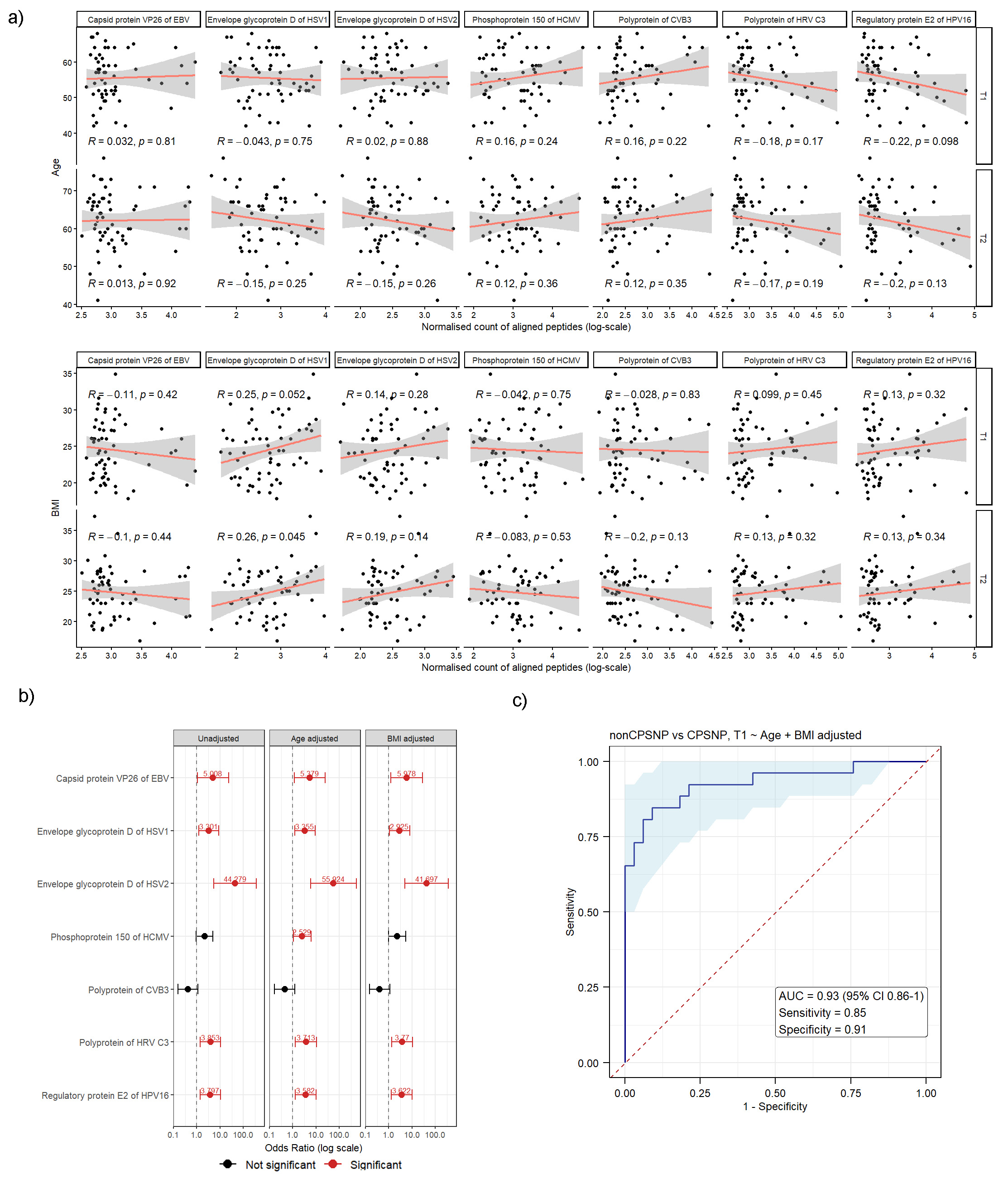


***Figure S6.*** *Relationships between the values of antibody response to 7 pathogen epitopes and patients age and BMI during surgical nerve injury (T1) or during follow-up (T2). a) Scatter plots show the relationship between age or BMI and log-transformed normalised counts of aligned peptides for each antigen in both timepoints (T1 and T2). The solid line represents the fitted linear regression. Pearson’s correlation coefficient (R) with 95% confidence intervals is shown in each panel. b) Association between epitope-specific log-transformed normalised peptide counts and CPSNP status in T1 samples. Separate multivariable logistic regression models were fitted for each antigen, with CPSNP status as the binary outcome and epitope-specific normalised counts as the primary predictor, adjusting for age and BMI. Odds ratios (ORs) with 95% confidence intervals are shown on a logarithmic scale. The vertical dashed line indicates an OR of 1 (no association). Statistically significant associations (p < 0.05) are highlighted in red and OR values are provided. c) Receiver operating characteristic (ROC) curve for discrimination between CPSNP and non-CPSNP cases in pre-operative samples. A multivariable logistic regression model incorporating five epitope-specific log-transformed normalised peptide counts (pp150 of HCMV, gpD of HSV2, E2 of HPV16, VP26 of EBV and polyprotein of CVB3) and age and BMI contribution of T1 samples was used to predict case–control status. The ROC curve shows sensitivity plotted against 1 − specificity. The shaded area represents the 95% confidence interval of the ROC curve estimated by bootstrapping (DeLong). The area under the curve (AUC) with 95% confidence intervals, as well as sensitivity and specificity at the optimal Youden index threshold, are displayed within the panel. Groups compared and presented on figure panels a-c: CPSNP, n=26; non-CPSNP + CTRL, n=29 +4.*

 
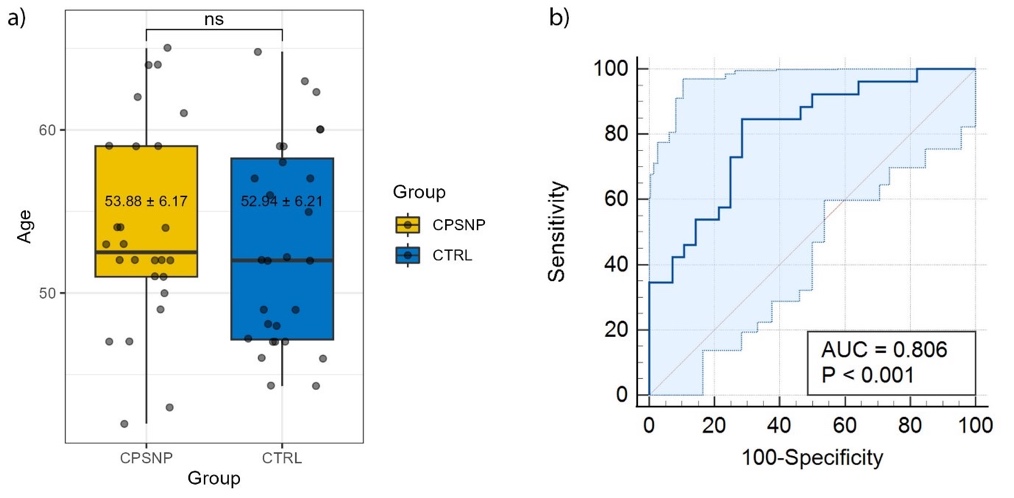


***Figure S7. Logistic regression model with the values of antibody response to 5 pathogen epitopes as validated against an independent control cohort (n=28). a)*** *Independent control cohort consisted of the immunoprofiles from samples of 28 age-matched healthy Finnish women (CTRL cohort described in PMID: 36207326, PMID: 35091341 when compared to 26 first timepoint CPSNP samples. Wilcoxon Rank Sum test, p-values unadjusted: ns p> 0.05.* ***b)*** *Logistic regression model comparing 26 CPSNP and 28 CTRL sample immunoprofiles of antibody response to pp150 of HCMV, gpD of HSV2, E2 of HPV16, VP26 of EBV and polyprotein of CVB3. Sensitivity = 0.73, Specificity= 0.75, AUC = 0.806. Light blue area shows the 95% CI of sensitivity and specificity.*

***Table S1. Clinical characteristics of 63 breast cancer patients and their immunoprofile description.*** *A total of 63 breast cancer patients, each providing samples at two time points (T1 and T2), were included in the MVA immunoprofiling analysis. In total, 126 samples were immunoprofiled, and serological measurements for EBV, HCMV, HSV1, and HSV2 were conducted using the T1 samples from all 63 patients. We selected the 5000 most abundantly detected peptides, based on IgG binding values (read counts), from each sample for the immunoprofile similarity analysis. To facilitate comparisons, we calculated the normalized scalar products of the peptide count vectors to derive the cosine similarity index using the "lsa" R package, resulting in a 126x126 sample immunoprofile similarity matrix. The column labeled "CSI_with_unpaired_Sample" indicates the maximum similarity value for each sample when compared to another unpaired cohort sample. A threshold of CSI < 0.7 was applied, leading to the exclusion of 4 samples (2 controls, 1 CPSNP patient, and 1 non-CPSNP patient) from the subsequent group comparisons and regression analyses (SampleInAnalyses). For clarity, the timepoint sample used in each figure for each sample is shown.*

***Table S2. Annotation of infections and antigens associated with CPSNP using epitope sequences from IEDB.*** *1882 group discriminative epitopes were aligned to the full export of Immune Epitope Database (IEDB, version 3, exported on 15.04.2024, containing 2,225,695 epitopes). To evaluate the statistical significance of specific epitope–motif alignments, we performed a permutation-based test for each aligned epitope–motif pair (n=6254). For each pair, the epitope sequence was held constant while the amino acid positions of the matched motif were randomly shuffled 10,000 times to generate permuted motif variants preserving amino acid composition. Each permuted motif was aligned to the original epitope sequence, and the number of exact matches was recorded. The empirical p-value was calculated as: (number of permuted matches +1) / (number of permutations + 1) and p-values were adjusted with FDR. Full alignment results were cleaned and altogether* *79 most relevant infection-associated pathogens related to top immune response were included in hit analysis. Altogether, the 1882 group discriminating epitopes aligned to 4188 epitopes of the antigens of 79 pathogens. The values of Student’s t-test and ROC values describing the discriminating power of CPSNP from non-CPSNP in both pre- and post-operative timepoints, and the location of each of the 1882 features in the 2-dimenstional tSNE plot are provided.*

***Table S3. Annotation of most relevant epitopes associated with CPSNP using infectious epitope sequences from IEDB.*** *All relevant infection-associated referenced epitopes related to the response of the adaptive immune system from the IEDB (Immune epitope database, epitope full export on 15.04.2024,* <https://www.iedb.org/>*) and 1882 group discriminative epitope mimics from MVA were included in the study. Seven pathogen epitopes (polyprotein of HRV C3; regulatory protein E2 of HPV16; phosphoprotein 150 of HCMV; polyprotein of CVB3; capsid protein VP26 of EBV) were deemed most interesting by the number of sequence similarity hits each epitope gained (****Table S2****). To evaluate the statistical significance of specific protein–motif alignments, we searched for epitope-aligned motifs from the parent proteins of the epitopes and performed a permutation-based test for each aligned protein–motif pair (n=116, 111 unique motifs). For each pair, the primary protein sequence was held constant while the amino acid positions of the matched motif were randomly shuffled 10,000 times to generate permuted motif variants preserving amino acid composition. Each permuted motif was aligned to the original protein sequence, and the number of exact matches was recorded. The empirical p-value was calculated as: (number of permuted matches + 1) / (number of permutations + 1) and p-values were adjusted with FDR.*

***Table S4. Annotation of autoantigenic epitopes associated with CPSNP using human epitope sequences from IEDB.*** *All autoimmune-associated referenced epitopes related to the response of the adaptive immune system from the IEDB (Immune epitope database, epitope full export on 15.04.2024,* <https://www.iedb.org/>*) and 1882 infection related epitope mimics were included in the study. Altogether 613 different human protein epitopes showed sequence similarity to 76 of the queried motifs (belonging to 274 unique proteins, total 655 hits). Alignment probability of each feature was assessed with 10,000 permutations of the aligned feature and shown as empirical and FDR-adjusted p-values. For the antigens recognized in the study cellular location were gained from UNIPROT database (column intracellular/extracellular, column features – cytoplasm, endoplasmic reticulum, cell membrane, secreted, cell junction, cell projection, chromosome, Golgi apparatus, lysosome, mitochondrion, nucleus, recycling endosome, NA – data not available). A large majority of the epitopes described were intracellular. Extracellular recognition was described for following antigens - Collagen alpha-3(VI) chain (UNIPROT ID P12111), Protein S100-A9 (UNIPROT ID P06702), Interleukin-6 (UNIPROT ID P05231), Laminin subunit gamma-1 (UNIPROT ID P11047), Sialic acid-binding Ig-like lectin 5 (UNIPROT ID O15389), Multiple epidermal growth factor-like domains protein 8 (UNIPROT ID Q7Z7M0), Leucine-rich repeat neuronal protein 4 (UNIPROT ID Q8WUT4), CD70 antigen (UNIPROT ID P32970), Cytokine receptor common subunit gamma (UNIPROT ID P31785), Low-density lipoprotein receptor (UNIPROT ID P01130), CD320 antigen (UNIPROT ID Q9NPF0), Interleukin-31 receptor subunit alpha (UNIPROT ID Q8NI17), CX3C chemokine receptor 1 (UNIPROT ID P49238), Adhesion G protein-coupled receptor A2 (UNIPROT ID Q96PE1), Platelet glycoprotein 4 (UNIPROT ID P16671), Angiotensin-converting enzyme 2 (UNIPROT ID Q9BYF1), Lymphocyte activation gene 3 protein (UNIPROT ID P18627). Associated pathogen epitopes and aligned protein domains (Features) are described.*
